# We Have More Steps Along the Path to Routine Cardiorespiratory Fitness Evaluation

**DOI:** 10.1016/j.jacadv.2024.101080

**Published:** 2024-07-31

**Authors:** Matthew Colna, Sarah Abou Alaiwi, Katherine A.A. Clark

**Affiliations:** Section of Cardiovascular Medicine, Yale School of Medicine, New Haven, Connecticut, USA

**Keywords:** advanced heart failure, cardiopulmonary exercise test, cardiorespiratory fitness

**Figure:**
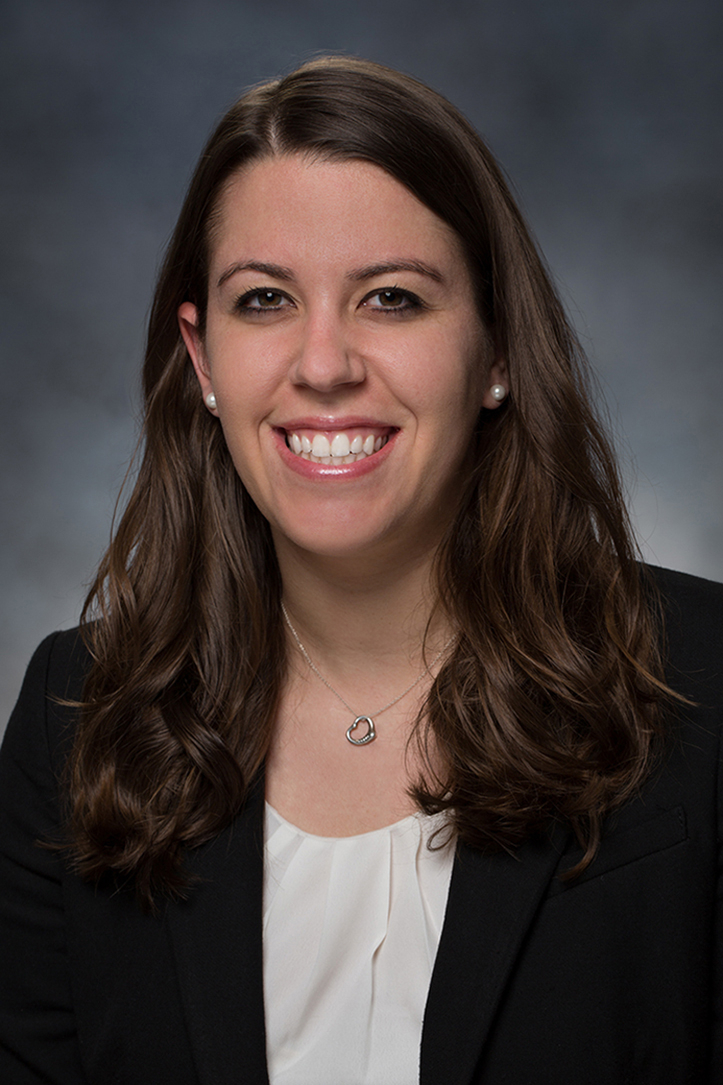

Cardiopulmonary exercise testing (CPET) currently stands as the benchmark method for directly evaluating cardiorespiratory fitness (CRF), which is an important measure of overall cardiovascular health and outcomes.^1^ CPET offers real-time analysis of 3 key variables in gas exchange: oxygen uptake (VO_2_), carbon dioxide output (VCO_2_), and ventilation (VE). These measures, alongside standard exercise metrics such as heart rate, blood pressure, electrocardiogram readings, cardiac imaging including echocardiography, as well as invasive hemodynamic assessments, can be employed during a CPET study to reveal specific patterns in gas exchange and recapitulate how organs respond to exercise in a maladaptive manner.^2^ Despite the immense amount of information that a CPET study can provide, its widespread use has been limited by accessibility due to competency requirements and high cost.^3^ Thus, incorporating portable and low-cost sensor devices into a simple bedside assessment of CRF could have the potential to improve the utilization and accessibility of this important prognostic evaluation.

In this issue of *JACC: Advances*, Molinger et al^4^ evaluated the association of a novel 6-Minute Incremental Step Test (6MIST) augmented with wearable sensor technology with the gold standard CPET evaluation, in a lauded effort to improve accessibility and facilitate evaluation of CRF. Briefly, the novel augmented 6MIST consists of an incremental pace stationary stepping exercise for a maximum of 6 minutes. To assess CRF, VO_2_ and VE were assessed using a portable VO_2_ analyzer; cardiac output was estimated using a portable, signal morphology-based impedance cardiograph with real-time wireless monitoring. However, due to the limitations of this technology, resting VO_2_ as well as respiratory exchange ratio (RER) and VE/VCO_2_ slope could not be obtained. The authors enrolled patients who were already scheduled to undergo a clinically indicated supine cycle ergometry CPET with invasive hemodynamics (iCPET) for a same day augmented 6MIST. To compare these methods of evaluating CRF, the correlation between hemodynamic parameters from both tests was assessed using the intraclass correlation coefficient. In total, fifteen patients (mean age 60 ± 14 years, 40% female, 27% Black) were included. All patients who agreed to undergo 6MIST completed the study without any test-related adverse events. The authors reported a good to excellent correlation between the iCPET- and 6MIST-measured CPET parameters including peak heart rate, absolute peak VO_2_, relative peak VO_2_, maximum VE, O_2_ pulse, and cardiorespiratory optimal point. No significant correlation was determined between iCPET and 6MIST in measuring cardiac index at rest or at peak exercise. Overall, while the authors clearly demonstrated the feasibility and correlation of a novel augmented 6MIST with wearable devices for simultaneous CPET and hemodynamic assessment, they note that additional studies are needed to confirm the validity of the 6MIST compared to standard upright CPET.

The authors have undertaken the noble and important task of improving upon a traditionally complex test that is of great diagnostic and prognostication value, by simplifying its execution on multiple fronts. The overarching goal of the augmented 6MIST test is to allow a CRF assessment to be more accessible to patients. To start, the authors employ a simple and very safe exercise test to obtain peak exercise capacity. Such a protocol would in theory be more practical for chronically ill patients to perform. Furthermore, utilizing a lower-cost, less-invasive wearable sensor technology would allow for the 6MIST to be performed more easily by a greater number of providers across a wider geographic distribution of health care centers, particularly compared to the current landscape of a small number of institutions at which iCPETs are performed. For example, the heart failure patient population in which these tests are primarily utilized could potentially benefit from a more timely assessment of their risk and prognostication to determine further treatment options and consideration for a more expedited referral to transplant and left ventricular assist device centers. In addition, such a test would perhaps allow more frequent repetition to evaluate a change in patients’ symptoms in order to more precisely ascertain subtle changes in their functional status at the bedside.

Although feasibility of the 6MIST was well established, a few limitations must be noted with the current technology and protocol. To start, this study had a rather small sample size of only 15 participants, which may have limited the statistical power of the intraclass correlation coefficient analysis. The authors did adapt an exercise protocol for the 6MIST from previously validated exercise protocol in a heart failure patient population, one must be cognizant that the protocols used between the iCPET and 6MIST were based on different types of exercise as the iCPET was supine while the 6MIST was upright, somewhat limiting the direct comparison between these 2 tests.^4^ Importantly, regarding the data obtained from the sensor technology, the gas exchange analysis during the 6MIST was limited by the lack of a CO_2_ sensor. Without the ability to analyze CO_2_, RER cannot be determined and defining anaerobic threshold is diminished, greatly inhibiting the diagnostic value of the 6MIST, especially in the patient population with advanced heart failure. In addition, the prognostic value of a CPET is only valid if a maximal effort is achieved as determined by the RER, again limiting the prognostic value of 6MIST.

Another consideration for implementation and generalizability, beyond the ability to perform the 6MIST, is that interpretation of the results would still require specialist-level expertise. Thorough exposure and training are not broadly and uniformly available in all cardiovascular or pulmonary critical care internal medicine clinical fellowship training programs due to the current landscape of CPET testing. Thus, although the 6MIST study would be simpler to execute, scaling the use of the 6MIST would still require growth in the population of trained interpreting physicians.

As we march forward, continued technological advances will no doubt improve the wearable sensor's ability to obtain robust clinical data in a less-invasive manner. As test validity is demonstrated, a next crucial step is how to best incorporate the 6MIST into clinical practice to maximize its impact on patient care. As the authors clearly state the 6MIST will not replace the CPET, further work is needed to define how it is added to various clinical scenarios and practice. In looking ahead, as these questions are answered, such a simplified bedside assessment of CRF would ideally improve patients’ outcomes. Despite the required upfront cost with scaling this technology, more expedited risk assessment, diagnosis, and ultimately treatment would have the concurrent potential of helping to defray health care resource utilization and costs.

Although the CPET will likely remain the gold standard for evaluating underlying drivers of dyspnea and determination of candidacy for advanced therapies for the foreseeable future, striving to incorporate novel more-portable less-invasive technologies is key to pushing the field forward to improve the care of patients with cardiovascular disease. Such advances in this type of CRF testing, once fully validated, have the opportunity to improve access and risk assessment to expedite the appropriate treatment. As a key first step toward this goal, this work has demonstrated the feasibility the 6MIST. Further work is needed to validate the 6MIST, specifically compared to the upright CPET, as well as understanding the clinical impact of routinely performing such a test.

## Funding support and author disclosures

The authors have reported that they have no relationships relevant to the contents of this paper to disclose.
